# Low whole grain intake in the UK: results from the National Diet and Nutrition Survey rolling programme 2008–11

**DOI:** 10.1017/S0007114515000422

**Published:** 2015-04-23

**Authors:** Kay D. Mann, Mark S. Pearce, Brigid McKevith, Frank Thielecke, Chris J. Seal

**Affiliations:** 1 Institute of Health and Society, Newcastle University, Newcastle upon TyneNE1 4LP, UK; 2 Human Nutrition Research Centre, School of Agriculture, Food and Rural Development, Agriculture Building, Kings Road, Newcastle University, Newcastle upon TyneNE1 7RU, UK; 3 Cereal Partners UK, Welwyn Garden CityAL7 1RR, UK; 4 Cereal Partners Worldwide, Lausanne, Switzerland; 5 Nestlé Research Center, Vers chez les Blanc, Lausanne, Switzerland

**Keywords:** Whole grain, Whole grain intake, UK population

## Abstract

Increased whole grain intake has been shown to reduce the risk of many non-communicable diseases. Countries including the USA, Canada, Denmark and Australia have specific dietary guidelines on whole grain intake but others, including the UK, do not. Data from 1986/87 and 2000/01 have shown that whole grain intake is low and declining in British adults. The aim of the present study was to describe whole grain intakes in the most current dietary assessment of UK households using data from the National Diet and Nutrition Survey rolling programme 2008–11. In the present study, 4 d diet diaries were completed by 3073 individuals between 2008 and 2011, along with details of socio-economic status (SES). The median daily whole grain intake, calculated for each individual on a dry weight basis, was 20 g/d for adults and 13 g/d for children/teenagers. The corresponding energy-adjusted whole grain intake was 27 g/10 MJ per d for adults and 20 g/10 MJ per d for children/teenagers. Whole grain intake (absolute and energy-adjusted) increased with age, but was lowest in teenagers (13–17 years) and younger adults up to the age of 34 years. Of the total study population, 18 % of adults and 15 % of children/teenagers did not consume any whole-grain foods. Individuals from lower SES groups had a significantly lower whole grain intake than those from more advantaged classifications. The whole grain intake in the UK, although higher than in 2000/01, remains low and below that in the US and Danish recommendations in all age classes. Favourable pricing with increased availability of whole-grain foods and education may help to increase whole grain intake in countries without whole-grain recommendations. Teenagers and younger adults may need targeting to help increase whole grain consumption.

Whole grains are defined as ‘the intact, ground, cracked or flaked kernels after the removal of inedible parts such as the hull and husk. The principal anatomical components the starchy endosperm, germ and bran are present in the same relative proportions as they exist in the intact kernel’^(^ ^1^ ^)^. The definition differs from the American Association of Cereal Chemists (AACC) International definition^(^ ^2^ ^)^ by allowing ‘Small losses of components, that is, less than 2 % of grain/10 % of bran that occur through processing methods consistent with safety and quality’^(^ ^1^ ^)^. A standardised definition of whole-grain foods has recently been proposed^(^ ^3^ ^)^, suggesting that a whole-grain food should provide 8 g of whole grain per 30 g serving in order to be defined as a whole-grain food. This recommendation was based on the authors' evaluation of available scientific literature which indicates that this amount of whole grain, without consideration of fibre content, is a minimum content of whole grains that improve diet quality sufficiently to result in health benefits.

The health benefits of consuming whole grains have been demonstrated in a large number of observational studies and in a number of dietary interventions. Together, these provide strong evidence for a reduction in the risk of several chronic diseases, notably CVD, type 2 diabetes, some cancers and an improvement in gut health^(^ ^4^ ^–^ ^12^ ^)^. The mechanisms through which whole grains provide health benefits are unclear, and may include the effects of reducing inflammatory status^(^ ^13^ ^)^, improving blood lipid profile and reducing or maintaining body-weight gain^(^ ^7^ ^)^, and lowering blood pressure^(^ ^14^ ^)^, as well as a variety of metabolic and hormonal effects attributed to an increased intake of phytochemicals^(^ ^15^ ^)^. These observed benefits have resulted in recommendations for intake and health claims in the USA^(^ ^16^ ^)^, although, to date, there have not been any claims approved by the European Food Safety Authority^(^ ^17^ ^)^. Recommendations for whole grain consumption vary among countries. For example, in the USA and Canada, the recommendation is that ‘all adults eat at least half their grains as whole grains – at least 3 to 5 servings of whole grains per day’^(^ ^18^ ^)^, building on the previous recommendation to consume three ‘ounce-equivalents’ of breads, rolls, cereals or other grain foods made with 100 % whole grains, or six ‘ounce-equivalents’ made with a mix of whole and refined grains^(^ ^19^ ^)^. In Denmark, the recommended intake of whole grain is higher at a minimum of 75 g of whole grains daily (per 10 MJ energy intake) or 60 g/d (per 8 MJ energy intake^(^ ^20^ ^)^). Food-based dietary guidelines in the WHO European Region^(^ ^21^ ^)^ recognise that quantification of portions and sizes of food-based guidelines are often unclear and difficult to interpret. Currently, no specific dietary recommendations for whole grain are present in the UK, other than recommending ‘choosing whole grain, brown or high fibre varieties wherever you can’^(^ ^22^ ^)^.

Previous studies of the UK diet have shown that whole grain intake is low and declining^(^ ^23^ ^,^ ^24^ ^)^. However, there has been an increase in whole-grain food products available in the consumer market, most notably in the USA^(^ ^25^ ^)^, and consumer awareness of whole grain is increasing, although barriers to their consumption still remain^(^ ^26^ ^,^ ^27^ ^)^. The last assessment of whole grain intake in the UK was undertaken by Thane *et al.* ^(^ ^23^ ^)^ presenting results of information collected in 1986–7 and 2000–1. The national dietary information from the UK has now been published from a collection period from 2008 to 2011 in the National Diet and Nutrition Survey (NDNS)^(^ ^28^ ^,^ ^29^ ^)^.

The aim of the present study was to quantify whole grain intake in the more recent diet of the UK population using data from the NDNS rolling programme 2008–11, and to assess variation in whole grain intake by socio-economic and demographic factors.

## Methods

### Study population

The NDNS is a nationally representative assessment of diet, nutritional intake and nutritional status of people aged 1·5 years and over, living in private households in England, Scotland, Wales and Northern Ireland. The methodology of the NDNS rolling programme including methods for food coding and nutrient analysis is described in detail elsewhere^(^ ^30^ ^,^ ^31^ ^)^.

Participants (*n* 3073, response rate of 55 % year 1 and 2, 52 % year 3^(^ ^30^ ^)^) completed an estimated food diary recording all food and drinks consumed both at home and away from home for four and, in some cases (*n* 53, 2 %), three consecutive days. Diary completion detail was explained to participants via a trained interviewer during the initial visit to their household along with an instruction booklet. Diet diaries for participants aged 11 years and younger were completed by a parent/carer with help from the child. Processing of the diet diary data was done by trained coders and editors. Food intakes were entered into the MRC HNR's (Medical Research Council, Human Nutrition Research) dietary assessment system, DINO (Diet In Nutrients Out). The food composition data used were from the Department of Health's (DoH) NDNS Nutrient Databank. Data coders matched each food/drink item recorded in the diary with a food code and portion code from DINO. Composite items (e.g. sandwiches) and home-made meals were split into their component parts and assigned individual food codes. Further details of data coding and editing are outlined in Appendix A of the NDNS official report^(^ ^30^ ^)^.

Social class was determined by the National Statistics Socio-economic Classification (NS-SEC)^(^ ^32^ ^)^ of the household reference person, defined as the householder (a person in whose name the property is owned or rented) with the highest income. If there was more than one householder and they had equal income, the eldest was selected as the household reference person. The NS-SEC classification is based on employment status of the household reference person at the time of the interview. This social classification is at the household level, not necessarily the individual who completed the diet diary. The NS-SEC contains eight classification groups with 1 assumed to be the most advantaged and 8 assumed to be the least advantaged; 1– higher managerial and professional occupations, 2 – lower managerial and professional occupations, 3 – intermediate occupations, 4 – small employers and own account workers, 5 – lower supervisory and technical occupations, 6 – semi-routine occupations, 7 – routine occupations, 8 – never worked.

The NDNS was conducted according to the guidelines laid down in the Declaration of Helsinki, and ethical approval for all procedures was granted by Local Research Ethics Committees covering all areas covered in the survey. All participants gave informed consent.

### Estimating whole grain intake

Of the 3659 foods consumed across the survey period, 221 foods were identified as containing any whole-grain ingredient. Whole grains considered in the present study follow those defined as whole grain in the publication by Seal *et al.* ^(^ ^33^ ^)^ and include whole wheat, wholemeal flour, wheat flakes, whole-grain wheat, whole and rolled oats, oatmeal, oat flakes, oat flour, brown and red rice, wild rice, whole-grain rice, rye flour, whole-grain rye, whole barley, whole corn/maize, popcorn, whole millet and quinoa. The whole-grain foods identified were sorted into nine easily distinguishable food commodity groups: bakes; bread; pasta; porridge; ready-to-eat cereals (RTEC); rice; savoury snacks; sweet snacks; other cereals. Bakes included buns, cakes, dumplings, tarts, sponges and scones, all made with whole-grain flours. Savoury snacks included crispbreads, crackerbreads, crackers, tortilla chips and crisp-like snacks. Sweet snacks included biscuits, cereal bars, popcorn and yoghurts with whole-grain cereal toppings. Other cereals included barley, oats, millet, quinoa and rye cereals.

The whole grain percentage, on a DM basis, for each whole-grain food identified was obtained from a list of whole-grain foods consumed by the British population^(^ ^34^ ^)^. A small number of whole-grain foods identified, which could not be found in this list, were obtained and whole grain percentage was calculated using the method described by Jones^(^ ^34^ ^)^. Where possible, weight losses of foods from processing/cooking were taken into account when estimating the percentage of whole grain content. For example, wholemeal bread, when toasted, loses 14·6 % weight during cooking^(^ ^35^ ^)^.

The method for quantifying absolute whole grain intake initially identified all whole-grain foods consumed by each survey participant. The whole grain content was calculated by multiplying the gram intake of each food identified by the percentage of whole grain. This was averaged over the number of food diary days recorded to give the estimated whole grain intake (g/d). According to the Dietary Guidelines for Americans 2010^(^ ^18^ ^)^, foods with at least 51 % whole grain contain a substantial amount of whole grain; therefore, a cut-off point of foods containing ≥ 51 % whole grain was also used and subsequent whole grain intake was calculated. A further cut-off point for foods containing ≥ 10 % whole grain was also considered for comparison with previously reported British whole grain intakes^(^ ^23^ ^,^ ^24^ ^)^. Whole grain intake is also reported in servings per d. A serving of whole grain was defined as 16 g/d in line with the US dietary guidelines 2010^(^ ^18^ ^)^, where 3 servings (‘ounce-equivalents’) are equivalent to 48 g of whole grain^(^ ^19^ ^)^. To account for differences in diet quantity by age and sex, whole grain intake was also adjusted for daily energy intake (10 MJ/d) as reported in the food diary.

### Data weighting and statistical analyses

Data used in the analyses were weighted in order to remove any potential selection bias in the observed results arising from non-response bias in the NDNS. Weighting variables to account for any potential bias in households, main food provider, individual selection, seasonality and for age, sex and regional profiles of participating individuals were provided by the NDNS team. Full details of weight computation are described elsewhere^(^ ^36^ ^)^. Whole grain intake and energy-adjusted whole grain intake were treated as continuous variables. Age, sex, whole grain serving and social classification were treated as categorical variables. Variation in whole grain intake was investigated by age, sex and social classification. Whole grain intake is reported as a median g/d or median g/10 MJ of energy per d with corresponding interquartile ranges (IQR) because the data were not normally distributed. A linear trend of whole grain intake by age was tested using linear regression. The Mann–Whitney rank sum test was used to test sex differences in whole grain intake, and the Kruskal–Wallis test was used to ascertain significant differences of whole grain intake by social classification. *P*< 0·05 was used to indicate significance throughout all statistical analyses, but actual *P* values are reported in tables.

All statistical analyses were done with Stata version 12 (Statacorp) using the complex survey functions.

## Results

### Whole grain intake

Of the 3073 food diaries completed, 1571 were completed by adults (age 18+ years) and 1502 were completed by children/teenagers (age 1·5–17 years). The population included 44 % male adults and 51 % male children/teenagers.

**Fig. 1:**
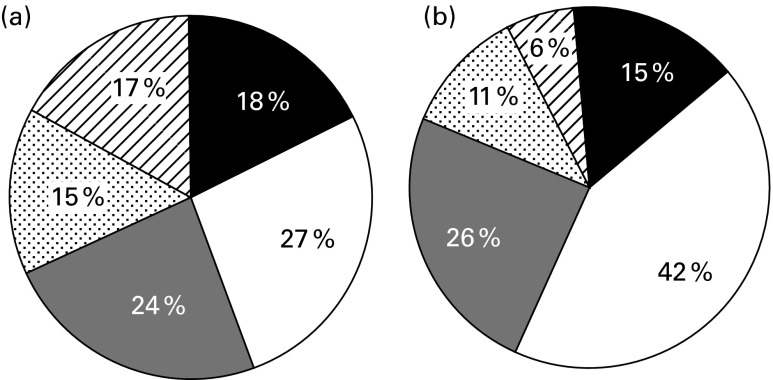
Proportion of (a) adult's (age 18 years+) and (b) children/teenager's (age 1·5–17 years) whole grain intake by serving (one serving equivalent to 16 g/d). ■, 0 g/d; 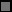, 16 to < 32 g/d (1 serving); 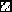, 48 g/d or more (3 servings); 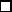, 0 to < 16 g/d; 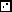, 32 to < 48 g/d (2 servings).

The median whole grain intake of the total population was 20 (IQR 5–39) and 13 (IQR 4–26) g/d for adults and children/teenagers, respectively. Of the whole population, 18 % of adults and 15 % of children/teenagers did not consume any whole-grain foods (Fig. 1). A whole grain intake of one 16 g serving/d was not achieved by 45 % of adults and 57 % of children/teenagers. Only 17 % of adults and 6 % of children/teenagers achieved a whole grain intake, which met the US dietary recommendation of 3–5 servings/d of whole grain.

**Fig. 2:**
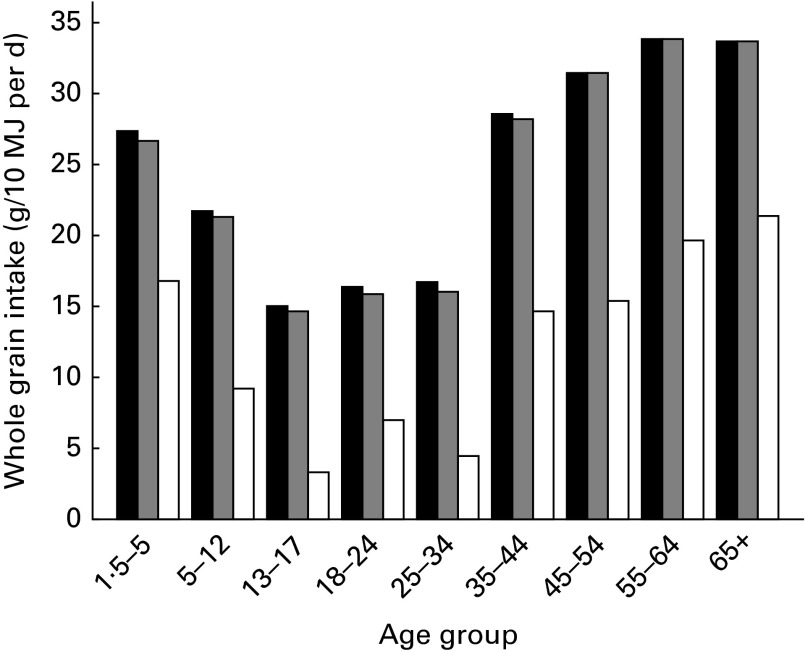
Daily median energy-adjusted whole grain intake by age group for foods with any whole grain content (■), foods with ≥ 10 % whole grain content (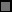) and foods with ≥ 51 % whole grain content (□).

The energy-adjusted median whole grain intake of the total population was 27 (IQR 6–52) and 20 (IQR 6–39) g/10MJ per d for adults and children/teenagers, respectively (Table 1). The median intake of whole grain ranged from 15 to 34 g/10 MJ per d across age groups, with the smallest intake in those aged 13 to 17 years (Fig. 2). Overall, whole grain intake significantly increased with age (*P* value for linear trend < 0·001); however, the intake of teenagers (13–17 years) and younger adults up to the age of 34 years was lower than all other age groups (Table 1; Fig. 2). In the oldest age grouping, 65+ years, there was a small decline in the median daily intake compared with the previous age group (55–64 years), but this was not significant.

**Table 1:** Energy-adjusted whole grain intake by sex

	Median energy-adjusted whole grain intake (g/10 MJ per d)	
	All whole-grain foods	≥ 10 % Whole-grain foods	≥ 51 % Whole-grain foods	
Age	Male	Female	All	Male	Female	All	Male	Female	All	*n*
Children/teenagers (years)										
1·5–5	28·1	26·8	27·3	28·1	25·8	26·8	16·3	17·0	16·8	484
5–12	25·0	18·6	21·7	24·3	17·4	21·3	10·7	8·0	9·3	577
13–17	15·1	14·9	15·0	14·7	14·1	14·7	4·4	2·0	3·3	441
Total	22·2	19·1	20·3	21·7	18·5*	19·6	10·4	8·0	9·3	1502
Adults (18+ years)										
18–24	10·6	17·4	16·3	10·6	17·4	15·9	5·9	12·1	7·0	194
25–34	18·1	14·9	16·7	18·1	14·6	16·0	8·4	4·1	4·5	228
35–44	29·4	27·8	28·5	28·5	17·8	28·2	13·2	18·6	14·7	311
45–54	21·5	34·9	31·4	21·5	34·9	31·4	12·7	19·2	15·4	275
55–64	36·3	33·7	33·8	36·6	33·7	33·8	19·9	19·7	19·7	258
65+	29·2	37·2	33·6	28	37·2	33·6	16·2	22·3	21·3	305
Total	23·2	29·6*	26·7	23·2	29·4*	26·6	12·7	16·8*	14·7	1571
Whole population	22·7	22·8	22·8	22·5	22·4	22·4	11·5	12·7	12·3	3073

* Value was significantly different between sex (*P*< 0·05; Mann–Whitney test).

There were negligible differences in the median whole grain intake (27 g/10 MJ per d adults, 20 g/10 MJ per d children/teenagers; Table 1) when intake was estimated considering only foods containing ≥ 10 % whole grain compared with all whole-grain foods (Table 1; Fig. 2). However, when estimating whole grain intake only from foods containing ≥ 51 % whole grain, median daily intakes were much lower. When using this cut-off value, the median intakes were 15 (IQR 0–38) and 9 (IQR 0–28) g/10 MJ per d for adults and children/teenagers, respectively, and ranged from 3 to 21 g/10 MJ per d across age groups, again with the lowest intake in those aged 13–17 years (Table 1; Fig. 2). Of the whole population, 35 % of adults and 40 % of children/teenagers consumed no foods containing ≥ 51 % whole grain.

The unadjusted median whole grain intakes of all foods and foods with ≥ 10 % whole grain content were 20 (IQR 3–42) and 19 (IQR 6–35) g/d for males and females, respectively, which were not significantly different. For child/teenage males and females, the median intakes were significantly higher for males compared with females (*P*< 0·001) at 15 (IQR 5–29) and 11 (IQR 4–22) g/d, respectively. Considering foods with 51 % or more whole grain content, the median daily whole grain intake of adults was reversed with females having higher intake than males, although this difference in intake was not significant. Whole grain intake from foods containing ≥ 51 % whole grain was significantly higher in children/teenager males, 7 (IQR 0–20) g/d, compared with females, 5 (IQR 0–16) g/d (*P*= 0·025). After adjustment for total energy intake, a significant difference (*P*= 0·002) was seen in adult females, 30 (IQR 9–55) g/10 MJ per d, compared with males, 23 (IQR 4–50) g/10 MJ per d (Table 1). In children/teenagers, the adjustment for energy intake removed the significant difference between sexes.

Details of social class were available for 3008 (98 %) of the participants completing a diet diary. The median daily whole grain intake of all foods increased by social classification (*P*< 0·001), with highest intakes in the most advantaged social classes and smallest in the lower two classes (routine occupations and never worked; Table 2). Each NS-SEC classification contained absolute non-consumers of whole grain with 9 % absolute non-consumers in class 1 (high managerial and professional occupations) increasing up to 26 and 20 % in those in routine occupations and never worked, respectively. No differences in median intakes across NS-SEC classes were seen considering foods containing ≥ 10 % whole grain. Considering foods with ≥ 51 % whole grain, the trend across NS-SEC classifications was less varied between high managerial/professional, low managerial/professional, intermediate, small employers, lower supervisory/technical and semi-routine occupations. At this cut-off point, the median whole grain intake was 0 g/d for the lower two social classes (routine occupations and never worked) and ranged between 5 and 14 g/d across the remaining classes described above.

**Table 2:** Whole grain intake by socio-economic classification measured by National Statistics Socio-economic Classification (NS-SEC)* (Number of subjects, percentages, medians, interquartile range (IQR); minimum (Min) and maximum (Max) values)

			Whole grain (g/d)
Social class using NS-SEC	*n*	%	Median	IQR	Min	Max
1. Higher managerial and professional occupations	446	15	24	10–40	0	220
2. Lower managerial and professional occupations	833	28	18	7–34	0	142
3. Intermediate occupations	256	8	17	4–31	0	158
4. Small employers and own account workers	330	11	15	3–30	0	157
5. Lower supervisory and technical occupations	327	11	15	5–34	0	156
6. Semi-routine occupations	404	13	14	2–29	0	181
7. Routine occupations	333	11	8	1–21	0	286
8. Never worked	79	3	8	1–22	0	99
All	3008	16	4 – 32	0	286

* Median whole grain intake distribution across all NS-SEC groups (*P*< 0·001; Kruskal–Wallis equality test).

### Sources of whole grain intake

Over the duration of the food diary recording (a total of 12 239 d), there were 6419 and 5561 whole-grain food-eating occasions for adults and children/teenagers, respectively (Table 3). In adults, the most frequently consumed whole-grain foods were whole-grain breads (44 %), followed by RTEC (27 %). In children/teenagers, the most frequently consumed whole-grain foods were RTEC (36 %), followed closely by whole-grain breads (35 %). Sub-dividing the population by age (Table 3) showed that the most frequently consumed whole-grain foods were RTEC in young children (age 1·5–5 years), whereas whole-grain bread was more frequently consumed in 5- to 17-year-olds. The contribution of RTEC to whole grain consumption declined with age. Sweet snack consumption was most prevalent in teenagers (age 13–17 years); savoury snack consumption was most prevalent in adults (age 18–24 years); porridge consumption was favoured more by the oldest (over 65 s); and whole-grain rice, pasta and bakes contributed less than 3 % of all whole-grain foods consumed.

**Table 3:** Percentage contribution of whole grain (WG) food groups by age to whole grain eating occasions

	Children/teenager age group	Adult age group	
WG food group	1·5–5	5–12	13–17	18–24	25–34	35–44	45–54	55–64	65+	Total
RTEC	38·7	34·7	32·3	33·6	28·1	26·8	27·7	26·3	24·5	31·0
Bread	35·6	35·2	35·1	41·1	42·7	44·7	44·5	46·3	41·1	39·7
Sweet snacks	11·7	19·8	21·0	10·9	15·8	12·5	11·1	10·7	13·7	14·6
Porridge	7·0	4·0	2·4	2·9	3·0	5·4	5·8	7·4	10·6	5·8
Savoury snacks	1·8	2·5	5·5	7·1	3·6	4·4	5·2	3·2	4·3	3·7
Other cereals	3·2	1·8	1·5	1·5	2·6	3·9	3·6	4·0	4·4	3·0
Rice	0·9	0·6	1·6	1·5	3·7	1·5	1·0	1·0	0·7	1·2
Pasta	1·0	1·3	0·6	0·6	0·1	0·7	0·5	0·9	0·2	0·8
Bakes	0·1	0·1	0·0	0·8	0·4	0·1	0·6	0·2	0·5	0·2
Consumers (*n*)	426	514	335	143	167	263	233	218	270	
Total	1275	1294	2569
Total number of WG eating occasions	2109	2232	1220	479	727	1196	1166	1197	1654	
Total	5561	6419	11 980

RTEC, ready-to-eat cereals.

Across all foods identified, the main whole grain consumed was wheat, accounting for 77 % of the overall whole grain consumption coming from a variety of foods, mainly bread (63 %) and RTEC (32 %). Oats accounted for 15 % of all foods consumed occurring in porridge (32 %), RTEC (26 %), as an ingredient in other cereal foods (25 %) and in sweet snacks (15 %). Maize consumption (3 % of total) was consumed from savoury snacks (47 %), sweet snacks (33 %) and RTEC (20 %). The remaining whole grains (rice, rye, barley, quinoa and millet) accounted for 2 % or less each of overall whole grain consumption. When considering only foods with 10 % or more whole grain, the relative proportions of the grain types consumed were not affected. When only considering foods with 51 % or more whole grain content, the proportion of grain types consumed were 81 % wheat, 12 % oats, 4 % maize, 2 % rye, 0·7 % barley and 0·3 % rice.

## Discussion

The present study reports recent whole grain intake in the diet from a representative survey of UK adults and children/teenagers. The median intakes were 20 and 13 g/d for adults and children/teenagers, respectively, with 18 % of non-consumption in adults and 15 % of non-consumption in children/teenagers. Whole grain intake increased with age, differed by sex and increased by socio-economic status with higher intakes in the more advantaged classifications and in females. Whole grain intakes from foods containing ≥ 10 % whole grain were not dissimilar to foods with any whole grain content; however, intakes were lower and less varied when only considering foods with ≥ 51 % whole grain.

The reported whole grain intake in this survey is low, with the maximum median daily whole grain intake (24 g/d) reported in this cohort falling significantly below the US dietary recommendation of at least 3 servings/d (equivalent to 48 g/d^(^ ^18^ ^)^). Comparable populations, including in the USA, also report that average intakes do not meet this target^(^ ^37^ ^–^ ^39^ ^)^ and are approximately 1 serving/d (16 g), which 55 % of adults in the NDNS achieve. Whole grain intakes for children/teenagers in the present analysis are lower than recently reported for Irish children/teenagers^(^ ^40^ ^)^, whereas median whole grain intakes, on a wet weight basis, were 12·7 g/d for children and 13·4 g/d for teenagers, approximately 2·5 and 1·2 g higher, than the corresponding values seen for the NDNS cohort. The reasons for the difference are unclear, and further explanation of food pattern consumption for the two cohorts is warranted to identify foods consumed in Ireland but not in the UK.

Previous analyses of whole grain intake in the NDNS^(^ ^23^ ^,^ ^24^ ^)^ considered only foods containing ≥ 10 % whole grain. In these analyses of adults in 1986–7 and 2000–1 and of young people (age 4–18 years) in 1997, the median whole grain intakes were 16, 14 and 7 g/d, respectively. The present analysis suggests that there has been a small increase in whole grain intake in the UK population. This appears not to be attributable to the extra foods with < 10 % whole grain included since the average intakes of adults and children/teenagers from all whole-grain food sources do not differ from the average whole grain intake from foods containing ≥ 10 % whole grain. In the present analysis, 221 foods were identified as containing any whole grain ingredient. In 1986/7 and 2000/1, 196 and 153 whole grain foods were identified containing at least 10 % whole grain^(^ ^23^ ^)^. This may, in part, contribute to the small increase in whole grain intake seen in this population. However, in all three analyses, similar foods are coded as unique items. For example, branded RTEC are coded separately to supermarket brand RTEC, so the apparent increase in variety of whole-grain foods may be misleading. In contrast to the modest increases in the UK, whole grain intake in Denmark has increased markedly by 72 % from a population average of 32 g/d in 2000–4 to 55 g/d in 2011–2^(^ ^41^ ^)^ following the Danish national campaign to promote whole grain intake. The proportion of Danes meeting the Danish target of 75 g/10 MJ rose from 6 to 27 % of the population.

It is important to note some methodological differences between the analysis reported in the NDNS rolling programme and the previous analysis of the NDNS that may also account for the apparent increase in whole grain intake. First, since 2008, the NDNS has been conducted as an annual rolling programme, whereas previous NDNS were run as a series of cross-sectional studies. In the cross-sectional NDNS, diet dairies were recorded over 7 d, whereas in the rolling programme, diaries are recorded over only 4 d. Differences in the number of recording days have little effect on comparisons of average consumption of food groups or mean nutrient intakes; however, caution should be taken when comparing percentages of food group consumption and meeting dietary recommendations between the present analysis and that of the previous cross-sectional NDNS^(^ ^30^ ^)^. Finally, the diet diaries in the previous NDNS were weighed diaries, whereas in the rolling programme, estimated weights and quantities were used^(^ ^30^ ^)^.

No significant difference in median whole grain intakes for adult males and females was observed when data were unadjusted for energy intake. However, when adjusting for energy intake, a significant difference was present with a higher intake reported in females, suggesting a greater importance for whole-grain foods in the diets of women once the expected higher total energy/food consumption in males is accounted for. In children/teenagers, the reverse was seen with significantly higher whole grain food consumption in younger males than younger females. Once adjusted for energy intake, the apparent difference was removed. These data confirm the higher total food consumption in boys compared with girls, but suggests that the overall pattern of whole grain food intake is the same for both sexes. These observations emphasise the importance of energy adjustment in describing whole grain intake between sexes, but also imply a change in eating habits with age where older females increase their consumption of whole-grain foods.

Whole grain intake significantly increased with higher socio-economic status. Socio-economic status measured by the NS-SEC is based on occupation; therefore, the increase in whole grain intake may be explained by income and possibly education. Those in a more advantaged socio-economic position may have a higher education and knowledge about whole-grain foods and health as well as the financial ability to purchase such foods, since whole-grain foods are often more expensive than their refined grain counterparts on offer^(^ ^42^ ^)^. This is similar to other studies where income and food cost have previously been identified as confounders of whole grain intake^(^ ^26^ ^,^ ^43^ ^)^ as well as a barrier to adherence of dietary guidelines^(^ ^27^ ^)^.

The majority of the 221 foods identified in this data set were RTEC, sweet snacks, breads and porridge. RTEC and breads are part of a traditional UK diet, with RTEC being a convenient breakfast meal particularly in children. Porridge is becoming a more popular breakfast meal particularly in the convenience and food to go market^(^ ^44^ ^)^, and is readily available in the appropriate form for the very young. The sweet snacks food group includes cereal bars that have had increasing product introduction^(^ ^45^ ^)^, although their whole grain content is typically low. The popularity of RTEC and breads in the UK are similar to other populations such as Irish children and teenagers where 44–59 and 14–27 % of foods consumed were RTEC and breads, respectively^(^ ^40^ ^)^. Adult food consumption habits in this UK population are also similar to those seen in the US population where 32 % and 30 % of whole-grain foods contributing to whole grain intake were breads and RTEC^(^ ^36^ ^)^.

Standard definitions on the amount of whole grain, which should be included in a product for it to be classified as a ‘whole-grain food’, do not exist. In 1999, the US Food and Drug Administration (FDA)^(^ ^46^ ^)^ defined ‘a whole-grain food as one that contains ≥ 51 % whole-grain ingredient by weight’ in order to establish a whole-grain health claim. Therefore, many whole grain studies have used this definition for whole-grain foods^(^ ^47^ ^)^. Ferruzzi *et al.* ^(^ ^3^ ^)^ recently proposed that whole-grain foods should provide 8 g of whole grain/30 g serving (27 g/100 g) without a fibre requirement. However, it is possible to consume large amounts of foods containing a smaller percentage of whole-grain ingredients, which will significantly contribute to total whole grain intake^(^ ^23^ ^)^. The consequences of using different cut-off points for inclusion of whole-grain foods are highlighted in the present analysis and in the previous NDNS analysis^(^ ^23^ ^,^ ^24^ ^)^. This raises issues of developing public health strategies for promoting whole grain intake where confusion may arise in consumers understanding the difference between ‘whole grain intake’ and ‘whole grain food intake’. For research purposes when investigating diet–disease relationships it is the former that is important to define clearly and accurately.

### Strengths, weaknesses and limitations

A limitation of the present study and any dietary assessment method is the misreporting of food consumption. A short 4 d dietary recording period and follow-up visits made to participants by trained interviewers helped to minimise misreporting. The data presented in the present report have all been weighted using variables provided by the NDNS team. Weighting the data should remove any bias occurring due to differences in the probability of households and individuals who were randomly sampled to take part in the survey. Weighting the data will also remove any bias from those who were selected to take part but did not respond or refused.

While making every effort to accurately source and calculate whole grain content of foods consumed, some assumptions made during calculation may lead to both small underestimations and overestimations of whole grain intake. Matching foods to similar products and vague or no detail on product packaging may also result in underestimation and overestimation. However, a strict inclusion and exclusion criteria of whole-grain ingredients and rigorous calculation was adopted to obtain the best possible estimate of whole grain intake.

The NDNS data used in the present report span a 3-year period during which food products may have changed or been re-formulated. For example, many RTEC have reduced salt content, and some ready meals may have lowered their fat content, affecting the percentage of other ingredients and potentially affecting apparent nutrient intake. There is no current database of whole grain content of whole-grain foods in the UK other than that prepared by Jones^(^ ^34^ ^)^. Foods that were not available in the list from Jones were sourced in 2013 in order to get the best estimate of whole grain content in that food. This may be adequate for new foods, but may not reflect older foods.

The whole grain content of foods consumed has been calculated as a DM percentage to give the most accurate estimate of whole grain intake and for comparison with published data, which are generally reported on a DM basis. Different whole grains have different amounts of water content; for example, wholemeal wheat is estimated to contain 14 % water and whole oat contains 8·9 % water^(^ ^35^ ^)^. Previous studies on whole grain intake have used both DM^(^ ^23^ ^,^ ^24^ ^)^ and wet matter^(^ ^37^ ^,^ ^38^ ^,^ ^40^ ^)^ to calculate whole grain intake, and in some cases, no information is provided. Currently, there is no standard practice as to whether dry or wet matter percentage is used, which makes direct comparison between studies difficult. Accounting for water content will give a better estimate of whole grain intake regardless of which whole grain has been consumed; thus, more accurate results are produced.

The NDNS data of 3073 participants are expected to have adequate statistical power for analysis. However, as with all statistical analysis, there remains the chance for error within multiple hypothesis testing. The large sample size of the present study helps to reduce the chance of error, and the results found are not unexplainable or inconsistent with other published studies.

### Conclusion

Whole grain intake in the UK remains low and below the US and Danish recommendations, although a small increase in intake was reported compared with the data from 2000/01. Teenagers and younger adults had particularly low whole grain intake, and this population group may need targeting to help increase whole grain consumption. Reducing the cost and further increasing the availability of whole-grain foods together with better educational awareness may help to increase whole grain intake in the UK and in other countries without whole grain dietary recommendations. Further investigation into the associated health benefits of whole grain intake in this population is needed.
